# Cellulosic-Based Conductive Hydrogels for Electro-Active Tissues: A Review Summary

**DOI:** 10.3390/gels8030140

**Published:** 2022-02-23

**Authors:** Esubalew Kasaw Gebeyehu, Xiaofeng Sui, Biruk Fentahun Adamu, Kura Alemayehu Beyene, Melkie Getnet Tadesse

**Affiliations:** 1 Key Lab of Science and Technology of Eco-Textile, Ministry of Education, College of Chemistry, Chemical Engineering and Biotechnology, Donghua University, Shanghai 201620, China; esubalewtsega@gmail.com; 2 Textile Engineering Department, Ethiopian Institute of Textile and Fashion Technology, Bahir Dar University, Bahir Dar 1037, Ethiopia; birukfentahun2009@gmail.com (B.F.A.); kuraalemayehu@gmail.com (K.A.B.); 3 Innovation Center for Textile Science and Technology, Donghua University, Shanghai 201620, China; 4 College of Textiles, Donghua University, Shanghai 201620, China; 5 Textile Chemical Process Engineering Department, Ethiopian Institute of Textile and Fashion Technology, Bahir Dar University, Bahir Dar 1037, Ethiopia

**Keywords:** conductive hydrogel, cellulose, tissue engineering, hydrogel design and characterization, electro-active tissues

## Abstract

The use of hydrogel in tissue engineering is not entirely new. In the last six decades, researchers have used hydrogel to develop artificial organs and tissue for the diagnosis of real-life problems and research purposes. Trial and error dominated the first forty years of tissue generation. Nowadays, biomaterials research is constantly progressing in the direction of new materials with expanded capabilities to better meet the current needs. Knowing the biological phenomenon at the interaction among materials and the human body has promoted the development of smart bio-inert and bio-active polymeric materials or devices as a result of vigorous and consistent research. Hydrogels can be tailored to contain properties such as softness, porosity, adequate strength, biodegradability, and a suitable surface for adhesion; they are ideal for use as a scaffold to provide support for cellular attachment and control tissue shapes. Perhaps electrical conductivity in hydrogel polymers promotes the interaction of electrical signals among artificial neurons and simulates the physiological microenvironment of electro-active tissues. This paper presents a review of the current state-of-the-art related to the complete process of conductive hydrogel manufacturing for tissue engineering from cellulosic materials. The essential properties required by hydrogel for electro-active-tissue regeneration are explored after a short overview of hydrogel classification and manufacturing methods. To prepare hydrogel from cellulose, the base material, cellulose, is first synthesized from plant fibers or generated from bacteria, fungi, or animals. The natural chemistry of cellulose and its derivatives in the fabrication of hydrogels is briefly discussed. Thereafter, the current scenario and latest developments of cellulose-based conductive hydrogels for tissue engineering are reviewed with an illustration from the literature. Finally, the pro and cons of conductive hydrogels for tissue engineering are indicated.

## 1. Introduction

Every year, millions of people lose tissue or organs as a result of accidents or illnesses [1]. Tissue or organ transplantation is used to treat these patients. This approach, however, is constrained by the lack of donors. To solve the problem of the severe shortage of organ transplants, intensive research work, a review [2], has been performed in the last four decades to develop artificial organs and tissue for diagnosis and research purposes. Cells and their extracellular subassemblies are used to develop biological tissues for body repair, primarily with bio-based material scaffolds. The scaffolds support cellular attachment and regulate tissue shape. Some of the strategies used to develop scaffolds were tri-dimensional textiles [3,4], aerogel [5,6], hydrogels [1], nanofibers [7,8], and composites [9]. In all cases, scaffolds should ideally be sufficiently porous to enable the growth of cells, nutritional diffusion, and physiologic waste extraction [10]; have adequate tensile strength and elasticity [11]; have controlled degradation [12]; and possess suitable chemistry for cell adhesion [13]. Other desirable properties, such as electrical conductivity, in polymers have also been reported to accelerate the nerve regeneration in artificial nerve grafts [14]. However, the importance of electrically conductive hydrogels in tissue engineering has received insufficient attention as yet [15].

**Figure 1:**
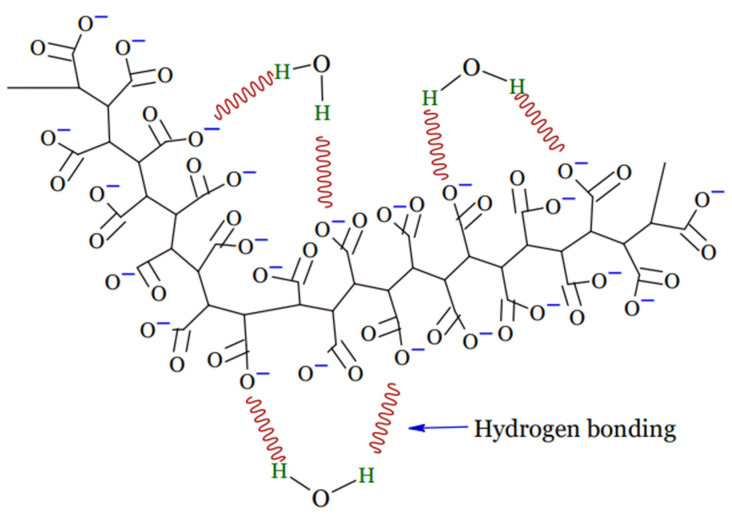
Structure of hydrogel.

A hydrogel is a tri-dimensional polymeric material that can take the form of a matrix, film, liquid, or microsphere [16] which is water insoluble and has the ability to swell and preserve a significant amount of water, typically greater than the mass ratio of the polymer materials in their interstitial structures [17]. Due to the presence of hydrophilic groups, such as –NH_2_, –OH, –COOH, and –SO_3_H, in their polymer networks and osmotic pressure, hydrogels continue to absorb and swell upon contact with water to form 3D structures. Physical or chemical crosslinking causes the ability of hydrogels to maintain an unaltered 3D structure during swelling, and this also helps to prevent hydrogels from dissolving in the solvent [18]. Upon hydration in an aqueous environment, hydrophilic groups or domains of polymeric networks form the hydrogel structure presented in Figure 1 [19].

**Figure 2:**
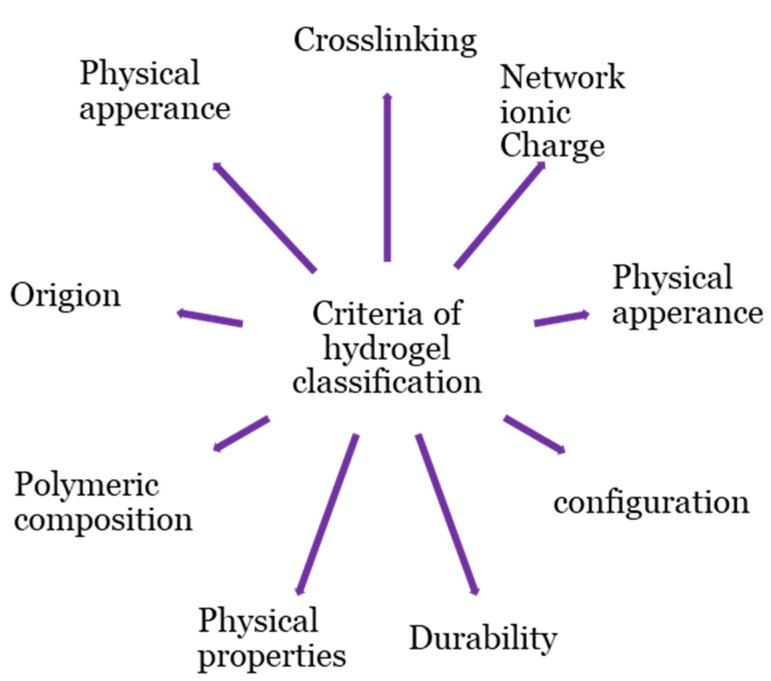
Classification bases of hydrogel.

Hydrogel products are classified based on a variety of criteria, as shown in Figure 2. The origins of the polymeric constituent of hydrogel can be classified as synthetic [20], hybrid [21], and natural [22]. Hydrogels from natural sources [22] can be derived from polysaccharide-based materials, such as cellulose [23]; chitosan [24]; glycosaminoglycans [25]; alginate [26,27]; protein-based materials, such as, silk fibroin [28,29], collagen [30], elastin [31], gelatin [32], and fibrin [33]; and decellularized hydrogels [34,35]. The integrity of the hydrogel is maintained through chemical crosslinking, physical crosslinking, or both [17,19,36,37,38]. Physical crosslinking, such as heating/cooling, hydrophobic interactions, freezing–thawing, complex coacervation, ionic interactions, and hydrogen bonding, have resulted in temporary networks of hydrogels, whereas permanent junctions exist in chemically crosslinked networks [16] obtained by employing grafting, chemical crosslinkers, radiation crosslinking, enzymatic reactions, click chemistry, radical polymerization, and thermo-gelation. A crosslinked network is a set of one, two, or three, and maybe more, types of monomers referred to as homopolymer, copolymer, and multipolymer, respectively [17]. Perhaps the hydrogel network is arranged in a network to swell up in a monomer; afterward, it reacts by forming a second intermeshing network structure to form interpenetrating polymeric composition [20].

The spatiotemporal control of hydrogel physicochemical characteristics is essential for monitoring their dynamics, such as durability and orientation [39]. The orientational dynamic appearance of biomolecules may be either crystalline or amorphous.

Hydrogels can be biodegradable or non-biodegradable in terms of durability [20]. Hydrogel physical properties have been advanced from conventional to smart [16]. Smart hydrogels are stimuli-sensitive and change the volume of the system structure in response to various stimuli, such as electric, light, temperature, and pH [17,40,41,42].

The work presented in this review aims to demonstrate the possibilities for producing conductive hydrogel of cellulosic-based materials. The organic compound cellulose was preferred for the hydrogel base material in this review, due to its excellent inherent properties, such as renewability [43,44], absorption ability [45], hygroscopicity [46], air permeability [47], biocompatibility [48,49,50], stability [51], bioactivity [50], and biodegradability [52]. In contrast to some of the other studies discussed in this review, however, design parameters’ consideration and characterization of hydrogel scaffolds for electro-active tissues, in general, and the preparation techniques of cellulosic-based conductive hydrogel, in particular, are given a place of due attention on this list.

## 2. Cellulosic-Based Hydrogel as Biomaterials

Biomaterials should be biocompatible and still have specialized biochemical, mechanical, and physical attributes in order to mimic fundamental aspects of the in vivo conditions. The need to assess the biocompatibility of biomaterial is to examine the biological responses that may cause harm or unwanted side effects to the host. In tissue engineering, biomaterials need to be biocompatible to have the ability to function as a support for tissue regeneration without eliciting any adverse local or systemic response in the eventual host. Biocompatibility is the potential of a material to coexist and interact in the presence of specific tissues or biological functions without causing excessive harm. The purpose of making a hydrogel biocompatible is to assess its toxic impact on the body [53]. Another attribute of a biomaterial is its bio-activity. A bioactive material is one that has been designed to induce specific biological activity, for example, tissue uptake, metabolism, or physiological response [54,55]. Cell arrangement, viability, and function are all influenced by biomechanical interactions between cells and biomaterial scaffolds. Biomechanics is the analysis of the interaction of biomedical scaffold and biological structures of cells, as well as the effects of such forces during stem-cell differentiation and molecular transport [56].

Tissue function in tissue engineering is affected by cell adhesion, proliferation, differentiation, and maturation. Biocompatibility, bioactivity, and biomechanics are critical requirements for any biomaterial, in general, and tissue engineering, in particular; cellulose-based biomaterials meet all three of these requirements. Cell adhesion facility, in conjunction with cellulose’s hydrophilic hydroxyl substituent, as well as specialized β-d environments, helps cells to attach to cellulose [57]. Due to the nature variety of chemical structures and functional properties, cellulose-based hydrogels are nontoxic. The patterned porosity of hydrogel scaffolds was found to promote the cellular adhesion, growth, proliferation, and infiltration of cells [58].

**Figure 3:**
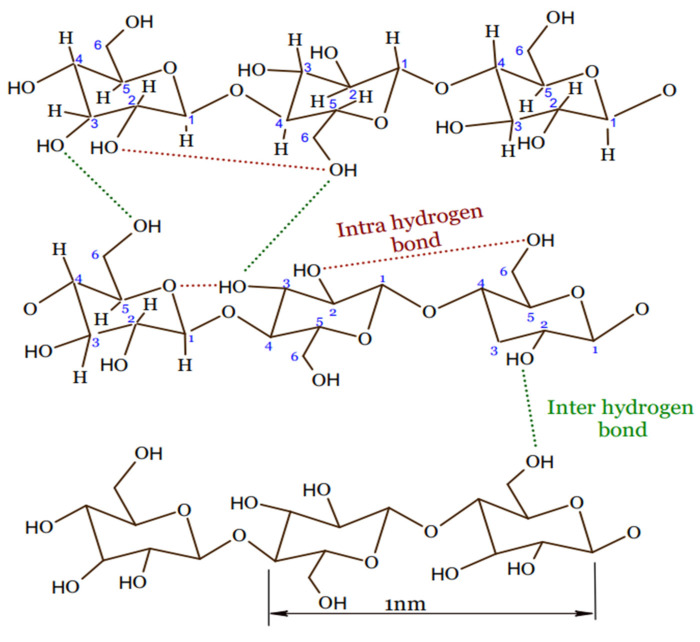
Cellulose structure.

Cellulose is a polysaccharide polymer that is made of a linear chain of glucose molecules [59]. Five thousand to fifteen thousand glucose molecules with the molecular formula (C_6_H_10_O_5_)_n_ are covalently bonded together to form cellulose by acetal oxygen via covalent bonding of C_1_ of glucose ring and C_4_ of the adjacent ring, as shown in Figure 3 [59,60].

The electrical characteristic of the body is essential in the physiological aspects of life [61]. Electric-potential stimulation occurs in a number of stem-cell functions, including cell interaction and the stimulation of signal transduction involved in cell-cycle progression. For instance, neurological rehabilitation and cell birth occur when nerve cells access the voltage-gated route liable for receptor activation in the presence of electrical activity, and cardiomyocytes are electrically active cells that generate contractile force when heart tissue works in tandem with bone tissue in a living heart. Even bone cells can be electrically stimulated as a result of the stress exerted on them by muscle contractions. Hence, the cells make use of electrical characteristics for a number of different purposes, and electrical stimulation of tissue in a controlled and targeted manner can enhance vascularization and differentiation of stem cells into different types of cells [61].

## 3. Classification of Cellulose Hydrogels

### 3.1. Source

**Figure 4:**
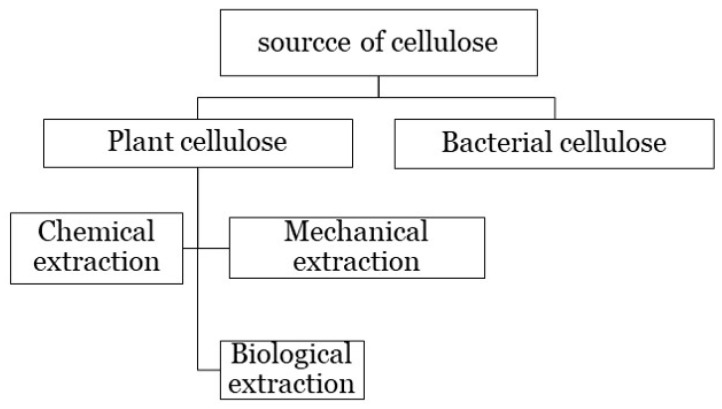
Cellulose source.

Cellulose is abundantly found in plant-cell walls or synthesis from bacteria (Figure 4) [57]. Cellulose is extracted from reinforcing the polymer of the cell walls of plants via chemical, mechanical, and biological extraction [62]. It is also synthesized from extracellular polysaccharides that are produced as protective envelopes around the cells of bacteria [38,63,64]. Diverse bacteria produce celluloses with varying morphological features, structures, characteristics, and functionalities [64]. Plant cellulose and bacterial cellulose differ in terms of macromolecular properties [65]. Plant cellulose contains impurities, such as hemicellulose and lignin; has a moderate water-holding capacity of 60%; and possesses a moderate level of tensile strength and crystallinity. Meanwhile, bacterial cellulose is chemically pure, which is hydrophilic and has a high water-holding capacity (100%), as well as high crystallinity and tensile strength [66]. A wide range of studies have been conducted on the difference of bacterial and plant cellulose as potential biomaterials [65,66].

To broaden its applicability, the esterification, etherification, and electrolytic dissociation reactions are used to modify the parent cellulose structure by substituting the hydroxyl group via organic species, such as methyl and ethyl units [23].

### 3.2. Crosslinking

**Figure 5:**
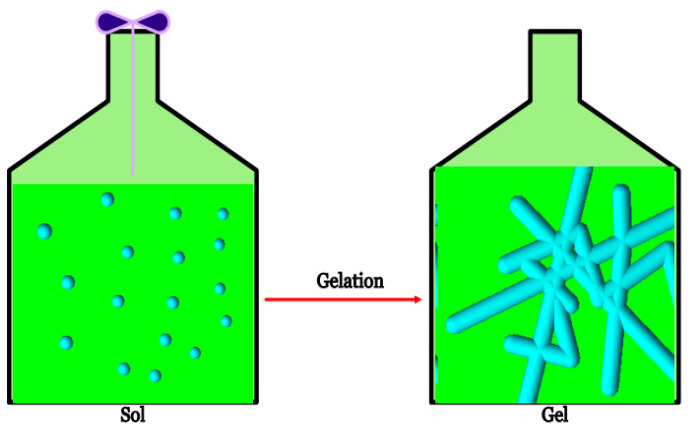
Sol–gel transition.

Cellulosic hydrogel scaffolds can be naturally derived or synthesized. The stabilization process of polymer to create the multidimensional extension of a polymeric chain to produce a network structure is achieved by performing either chemical or physical crosslinking. Linking of macromolecular chains together changes a liquid polymer into solid or gel. The rheological measurements of a liquid polymer is monitored by using the cone and plate geometry [67]. This transition from a structure with finite branched polymer to an infinite molecule system is referred to as gelation, and it results in an insoluble network [19,37,68] (Figure 5).

**Figure 6:**
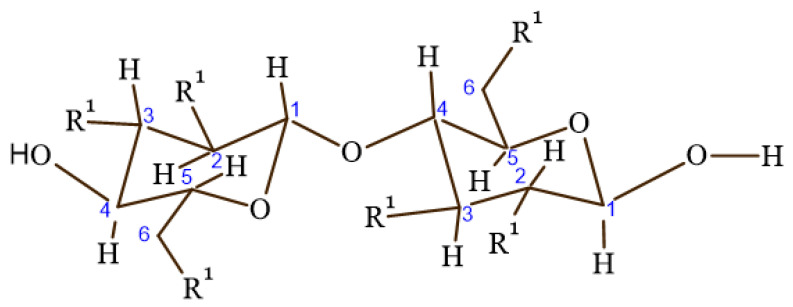
Chemical structure of ether derivatives, where R^1^ is OCH_3,_ OCH_2_CH_3,_ OCH_3_, [CH_2_CH_2_O]_n_H, and O[CH_2_CH(CH_3_)O]H OCH_2_COONa for MC, EC, HEMC, HPC, and CMC, respectively.

Permanent and temporary junctions of cellulose-based hydrogels can be manufactured by crosslinking aqueous solutions of cellulose derivatives, such as ethyl cellulose (EC), hydroxyethyl methylcellulose (HEMC), methylcellulose (MC), hydroxypropyl methylcellulose, Hydroxypropylcellulose (HPC), and sodium carboxymethylcellulose (CMC), which are the most common forms of etherified modified cellulose (Figure 6) [18,69]. A majority of water-soluble cellulose derivatives are produced through cellulose etherification, which occurs when the active hydroxyl groups of cellulose react with organic species such as methyl and ethyl units. The average number of etherified hydroxyl groups in a glucose unit determines the degree of substitution, which is controlled so that cellulose derivatives have the desired solubility and viscosity in water solutions [18]. The arrangement of functional chemical groups and their subsequent physicochemical characteristics are affected by the original material, as well as the fabrication method [57].

Due to the presence of numerous hydroxyl groups, which can link the polymer network via hydrogen bonding, hydrogels based on natural cellulose can be prepared from a pure cellulose solution via physical crosslinking [18,58]. The highly extended hydrogen-bonded structure of cellulose results in a compactness that is difficult to dissolve in common solvents. Hence, different solvents have been used to dissolve cellulose [58]. Table 1 shows a list of cellulose derivatives, as well as their solvents and processing methods.

## 4. Design and Characterization of Hydrogel Scaffolds

**Figure 7:**
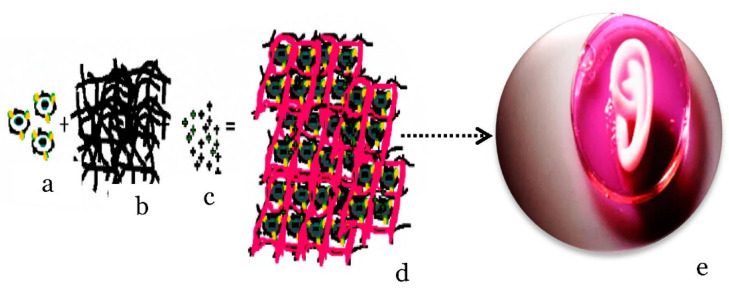
Tissue generation from cell to organ; cell (**a**), scaffold (**b**), bioactive factors (**c**), tissue engineering construct (**d**), and synthetic organ (**e**).

Tissue engineering integrates engineering and cell science principles and consists of three elements, namely scaffolds, cells, and growth factors, as shown in Figure 7 [36]. Scaffolds play an integral role in the development of tissue regeneration by providing structure support in tri-dimensional space to accommodate and guide their growth into a particular tissue [70]. Hydrogels can be used as scaffolds that imitate extracellular matrices to encapsulate and deliver cells, to provide structural integrity and bulk for cellular organization and morphogenic guidance, to act as tissue barriers and bio adhesives, to serve as drug depots, and to deliver bioactive moieties that promote natural reparative mechanisms [71]. Forming hydrogels for cellular experiments typically entails either encapsulating viable cells within the material or fabricating substrates, using molds that are later seeded with cells [72].

A variety of hydrogel properties, such as mechanics, swelling, mesh size, and degradation, may be of interest to characterize. When purchasing commercial kits or following specific hydrogel recipes, these may already be known and do not need to be described by every user. However, it is critical to understand how these characteristics are defined and how they may affect the utility of hydrogels in cell culture applications [72]. Hence, a proper designing of engineering hydrogel scaffolds considering all the possible factors is essential and a prerequisite for controlling cell orientation and tissue growth, and a few are listed in Table 2.

## 5. Hydrogel Conductivity Inclusion

### 5.1. Electro-Active Tissues

Electrical conductivity is an integral component of the human body [83]. Neurons function as a result of interacting networks woven by nerve cells. The nervous system is thought to contain approximately a trillion neurons. These highly irregularly shaped cells have the basic properties of the nervous system, such as intrinsic electrical conductivity. The ability of neurons to transmit signals from one neuron to another, as well as from a neuron to muscles and glands, is referred to as conductivity. The cell membrane allows a relatively large amounts of potassium ions to diffuse out of the cell, while allowing only a small amount of sodium ions to enter. These diffusive movements are simply the result of these ions moving down concentration gradients, following active transport by the sodium–potassium pump. When a voltage-gated ion channel opens, positively charged sodium ions diffuse into the axon, changing the membrane potential from −70 mv to zero and even higher, frequently reaching +35 mv. The membrane is said to have depolarized at that point. It happens in about a half-millisecond. The sodium gate then closes, and the usual outward diffusion of potassium occurs, causing the membrane potential to return to −70 and possibly lower to −73, due to a temporary overshoot in outward diffusion of potassium. This return to resisting is referred to as repolarization. Repolarization takes approximately half a millisecond. Thus, an action potential is a depolarization that is followed by a repolarization that takes about a millisecond to complete for a set of cells and tissues to function [55,84].

Electrical stimulation is a concern that is specific to a subset of cell types, including neurons and myocytes, in nerve-tissue engineering [85] (Table 3). As a result, electro-active biomaterials are required [61]. To meet these performance requirements, cellulose scaffolds coated with conductive materials can be used. Such materials have defined pore sizes, physicochemical characteristics, and electrical conductivities; they are also biocompatible and promote neurological differentiation [57].

### 5.2. Electro-Active Hydrogel

Cellulose scaffolds are an excellent material for nerve neurogenesis, due to their customizable surface chemistry and mechanical characteristics. Perhaps, to improve integrin-based attachment and cell–scaffold interactions, cellulose materials can be chemically modified and protein-coated [57]. Electro-active biomaterial-mediated stem-cell differentiation into specific cell lineages is of great significance for tissue regeneration. Although the underlying molecular events and mechanism of electro activation are not fully understood, there are some general guidelines for designing conductive hydrogels. Aside from matching the morphology and mechanical properties of hydrogels to the tissue microenvironment, it is critical to mimic the tissue electrophysiological environment. Neurons form synapses to transmit electrical signals and integrate into neuronal circuits in the mature nervous system. Neurons switch from an active electrical transmission state to an electrically silent and growth-competent state after axonal injury. When a cell shifts a single cell to multicellular collections and tissues, a striking parallel is found. Cells are regulated not only by their own potential, but also the potential of their neighboring cells via gap junctions [84,86].

Electrically conductive materials and crosslinked hydrogel networks are used to create conductive hydrogels through co-networks, blends, and self-assembly. This can be achieved through post-polymerization of a conducting monomer in a prefabricated hydrogel; composite strategies involving the mixing of conductive materials/monomers and hydrophilic polymers/monomers, followed by simultaneous or stepwise crosslinking to produce conductive hydrogels; and in situ polymerization involving the self-assembly of the modified electrically conductive materials [87]. The pros and cons of the strategies are given in Table 4.

Different types of conductive materials exhibit varying properties. Research (a review by Rong et al., 2018) [88] shows that three classes of materials are used on hydrogels for conductive purposes: metals, carbon allotropes, and intrinsically conductive polymers (ICPs).

For semi-conductor hydrogels, ionomers and silicones may be used as conducting materials as well [88]. As just an instance, when cellulose dissolved in an aqueous solution of benzyltrimethyl ammonium hydroxide (BzMe3NOH), ionic conductive cellulose hydrogels (CCHs) with anti-freezing properties were directly fabricated by chemical crosslinking without further treatment [89].

## 6. Incorporation of Conductive Materials on to Cellulose Hydrogels

### 6.1. Intrinsically Conductive Polymers (ICPs)

ICPs are conjugated polymers that have an extended delocalized system of ***π*** electrons that generally runs along the polymer backbone and is made conductive through doping [90]. The free motion of the loosely held ***π*** electrons within the unsaturated segments can open an electrical path for itinerant charge carriers. However, the changes in surface zeta potential and polymer surface properties, such as wettability and spatial conformation, can affect the cell behavior of electrical stimulation behavior [91]. It was subsequently understood that several polymers, such as polyacetylene, polypyrrole (PPy), polyaniline, polythiophene, poly (p-phenylene), and poly (3,4-ethylenedioxythiophene) polystyrene sulfonate (PEDOT/PSS), are conjugated polymers whose electrical conductivity is dramatically increased by doping. Doping involves the addition of a small amount of a chemical agent, which alters the electronic structure. The doping process, on the other hand, is reversible and involves a redox process.

Two major fabrication routes have already been investigated for the development of conductive polymer hydrogels by using ICP: gelation of CPs and hydrophilic polymers/monomers by self-assembly or the introduction of cross-linkable elements, as well as chemical oxidation; and electrochemical polymerization in a prefabricated hydrogel [88]. In a specific instance, X. Liang et al. developed a conductive hydrogel by polymerizing PPy through a prefabricated of chemically crosslinked microcrystalline cellulose (MMC) [73]. Gelation and chemical physical polymerization were employed after mixing the bacterial cellulose (BC) and PEDOT/PSS [92] to enhance the conductivity also.

### 6.2. Carbon Allotropes

Carbon-based materials are regarded as promising conductive materials for the fabrication of conductive hydrogels, due to their unique properties of high electrical conductivities, excellent environmental stability, and low production costs [88]. Materials that consist of only carbon atoms can have a wide range of conductivities, from the insulator diamond to conductors such as charcoal [93], carbon black (CB), graphene, and carbon nanotubes (CNTs) [94,95]. The level of conductivity will depend on the degree of delocalized electrons, thus making the graphitization and purity of the carbon compounds important factors [96]. Carbon-based biomaterials are commonly used as reinforcing agents in tissue-engineering applications to improve the mechanical performance and conductivity of the polymer matrix. Along with their unique mechanical properties, chemical stability, large specific surface area, and high electrical conductivity, graphene and carbon nanotube-based materials are the most widely used in tissue engineering. Furthermore, their large surface area and abundance of functional groups aid in the loading and release of bioactive species, such as chemical drugs, growth factors, genes, and proteins [83].

Blending with various polymers and self-assembly after modification are the two most common ways of preparing carbon-based conductive hydrogels [88]. Cellulose nanocrystals were grafted in to phenylboronic acid (CNCs-ABA) and multi-walled carbon nanotubes (MWCNTs) to develop electrical conductivity [97]. Another illustration is the post-polymerization of MWCNTs, with graphene powder (r-GOx) to adhere to pure regenerated cellulose-based electrolyte membranes [79].

Sometimes more than one conductive material may be used to enhance the conductivity of hydrogel. In a specific instance, to develop a conductive hydrogel, bacterial cellulose (rBC) slurry was mixed with PPy and single-walled carbon nanotubes (SWCNTs) and crosslinked in a stepwise manner. After preparing the rBC/PPy hydrogel, CNTs were added to the prepared rBC/PPy solution and dispersed before physical crosslinking had occurred [75]. The different preparation techniques of cellulosic-based conductive hydrogels is illustrated in Table 5.

### 6.3. Metals

Metals’ exceptional features, such as high conductivity, optical, magnetic, and catalytic properties, as well as metallic nanoparticles/nanowires, such as Al, Au, Ag, Cu, etc, have been widely used in the fabrication of conductive hydrogels [98]. Due to their high mechanical properties, fatigue resistance, and conductivity, bulk metals, such as titanium, magnesium, and stainless steel, have been used as bone-repair implants [83]. Although metals have some drawbacks, such as lack of flexibility, toxicity, cost, and negative environmental effects, they remain the only viable alternative for applications requiring high conductivity [91,99].

The common methods to develop metal-based conductive hydrogels are UV crosslinking and the in situ polymerization of hydrogel monomer and reduction of metal ions, using reducing agent [88]. An illustration of in situ polymerization through simultaneous crosslinking was performed by blending a precursor cellulose microcrystalline (CMC) solution; a monomer acrylic acid, initiator ammonium persulfate, catalyst N,N,N′,N′ tetramethylethylenediamine and crosslinker aluminum hexahydrate (AlCl_3_.6H_2_O), and the conductive materials of metallic ions of Al^3+^ produced conductive hydrogels [82]. Another example is grafting of acrylonitrile (AN) and acrylamide copolymers onto the hydroxypropyl methylcellulose (HPMC) chains in the presence of zinc chloride (ZnCl_2_), using ceric ammonium nitrate (AM) as the initiator [76]. In situ polymerization to form nanocomposite hydrogels of tannic acid–coated cellulose nanocrystal (TA@CNCs) ionic gel and then immersion in Al^3+^ solution to produce ionic coordination [100] have also been reported to develop cellulosic-based conductive hydrogel.

**Table 5:** Preparation techniques of cellulosic-based conductive hydrogel.

Hydrogel Features	Method of Crosslinking	Hydrogel Material	Conductivity (S/m)	(Potential) Application	Reference
Electro-active	Composite strategies	rBC/PPy and rBC/PPy/CNT	6.2 × 10^−2^	Cell proliferation	[75]
Conductive	Post-Polymerization	MCC/PPy	0.783	Electrochemical biosensors, electro-stimulated controlled drug release, and neural prosthetics	[73]
Conductive, self-healing, and strain- and thermal-sensitive performance	In situ polymerization	PAA-CMC-Al^3+^	162	Flexible and wearable temperature-sensing devices	[82]
Self-healing, shape memory, and biocompatible	Composite strategies	CNCs-ABA	3.8 × 10^−2^	Strain sensors	[97]
Ultra-stretchable, tough, anti-freezing, and conductive	Composite strategies via graft polymerization	HPMC-g-P (AN-co-AM)	1.54	Strain Sensor	[76]
Transparent, anti-freezing, and ionic conductive	Chemical crosslinking	CCHs	2.37	Sensor	[89]
Thermally stable, crystalline, and electroactive	Composite strategies	Polyvinyl alcohol cellulose (PC)		Actuator	[74]
Anisotropic and conductive, with high water content	Composite strategies	BC-PEDOT/ PSS		Scaffolds, implantable biosensors, and smart soft electronic devices	[92]
Tough, stretchable, self-adhesive, self-healing, and strain-sensitive	In situ polymerization	TA@CNCs	Conductivity is proved by light emitting diode	Wearable electronic sensors and healthcare monitoring	[100]
Electroactive and ultrafast for electro-mechanical response	Post-polymerization	Cellulose-based all-hydrogel artificial muscles membrane.	0.83–2.49	Transportation of nerve impulses from human muscle	[79]

## 7. Conclusions and Future Outlook

Tissue engineering and regeneration are growing fields that have the potential to revolutionize biomedical engineering. On the other hand, the translation of laboratory findings to the clinic has, indeed, been weak. The electrical stimulation of tissue can improve vascularization and differentiation of stem cells into various cells, but it is difficult to achieve targeted and controlled electrical stimulation. Electrically, conduction hydrogels hold great potential for conquering such barriers. In order to reconstruct completely operational tissue, the physicochemical and biological characteristics of hydrogel must enable cell generation and differentiation. The papers discussed in this article have consolidated research in the area of cellulose-based biomaterials in the broad sense of characteristics that regulate cellular functions and scaffold practicability for tissue engineering. Due to their diverse and adaptable physicochemical and biological properties, cellulose-based materials clearly have a high potential to become the next generation of standard biomaterials. Hydrogel properties are constantly evolving in an attempt to match the sophistication of native tissues.

In tissue construct, cell and tissue microenvironments vary at different periods throughout human life, notably during organ development and tissue repair, and designing an electro-active scaffold hydrogel that accommodate the changes over the period is a big challenge. Despite considerable advances in tissue engineering, neither material fully conveys the intricacies of native tissue or restores function to an ideal level. Conductive hydrogels have attracted a lot of attention for their widespread use in biomedical engineering, due to their structural similarity to soft tissue. However, designing conductive materials that combine biocompatibility with good mechanical and electrical properties remains difficult. AS a practical matter, the vestiges difficulty is just to develop new materials-design approaches in order to achieve actual biomimetic tissues. As the complexity of the application increases, such as in highly dynamic tissues, active remodeling of the scaffolding will be required. As a result, the complex interaction between cells and the artificial matrix will be critical. Perhaps cellulose-based hydrogels are difficult to prepare because cellulose is insoluble in water and common organic solvents, and the use of ionic liquid is evolving.

## Figures and Tables

**Table 1:** Summary of some cellulose derivatives and their corresponding hydrogel processing methods (copied from Reference [58]).

Cellulose and Cellulose Derivatives	Nature of Solvent	Solvent Systems	Corresponding Hydrogel Preparation Methods
Cellulose form wood	Polar solvents	NMMO	Solution polymerization at 85 °C
Cellulose from cotton pulp	Polar solvents	LiCl/DMAc	Solution polymerization at 75–90 °C
Filter paper	Ionic solvents	[Amim]Cl	Solution polymerization at 70 °C, 2 h
Tunicate cellulose	Alkali aqueous	Alkali/urea	Polymerization at −12 to −10 °C, 5–10 min
Cotton linter	systems	Alkali/thiourea	Polymerization at −5 °C, 2–10 min
Carboxymethylcellulose	Alkali aqueous	H_2_O	Solution polymerization, in situ polymerization
(CMC)	systems	DCM/DMSO	Solution polymerization, in situ polymerization
Methyl cellulose (MC)	Polar solvents	H_2_O	Solution polymerization, cryogenic treatment
Hydroxyethyl cellulose (HEC)	Polar solvents	H_2_O/ethanol	Solution polymerization, inverse-phase suspension polymerization
Hydroxypropyl methyl cellulose	Polar solvents	Acetone/ H_2_O	Chemical crosslinking

NMMO, *N*-methylmorpholine-*N*-oxide; LiCl/DMAc, lithium chloride/dimethylacetamide; [Amim]Cl, 1-allyl-3-methylimidazolium chloride; H_2_O, water; DCM/DMSO, dichloromethane/dimethyl sulfoxide.

**Table 2:** Design parameters consideration and characterization of hydrogel scaffolds for electro-active tissues.

Parameters	The Study Significance for Tissue Engineering	Instrument/Test Method	References
Molecular structures	To know the formation of hydrogel through investigating functional group reaction and intermolecular bonding.	Fourier-transform infrared (FTIR) spectroscopy	[73,74]
Morphologies	To justify suitability of hydrogel for cell adhesion by examining the mechanical toughness of hydrogel.	Scanning electron microscopy (SEM)	[73]
Polymer Morphology	To understand suitability of hydrogel for adhesivity to cells by studying the crystalline nature of polymeric hydrogels.	X-ray diffraction (XRD)	[73]
Cross polarization	Enables to know local magnetic fields around atomic nuclei/magnetic angle spinning by examining the molecular identity and structure.	Nuclear magnetic resonance (NMR)	[75]
Thermal stability	To Establish a connection between temperatures decomposition properties of substances through weight loss by studying the thermal property of the material.	Thermogravimetric (TGA) analysis	[73]
Thermal properties	To investigate the correlation between temperature and particular physical properties of the materials to use an aqueous phase diagram and the study of material physicochemical parameters in a composite formation.	Differential scanning calorimetry (DSC)	[74]
Swelling kinetics	Control of the most efficient way to transfer nutrients to cells and absorb wound exudates for rapid wound healing. The swelling properties can be used to detect batch-to-batch variations and consistency in hydrogel fabrication properties, as well as to determine whether the hydrogel mechanics are changing over time.	Soaking and swelling ratio calculation	[72,73,74]
Hydrophility	Enables to know the number of active hydrophilic groups.	Contact angle measurement by drop-shape analyzer	[75]
Electrical conductivities	Capable of delivering the electrical stimulation of nerve cells by measuring the electrical conductivity of scaffold.	Four-probe method, two-point probe, insulation resistance tester	[73,76,77]
Electrical and dielectric investigations	To investigate the correlation between temperature and electrical conductivity properties of the materials.	Broadband dielectric spectroscopy (BDS-40)	[78]
Electro mechanical properties	To simulate electrical properties of nerve cell by studying the dielectric behavior of gel through actuation test.	Laser displacement sensor	[74,79]
Mechanical properties	The durability and stability of the material in culture influence cellular mechanotransduction, which has consequences for cellular behaviors such as spreading, migration, and stem cell differentiation. It is investigated by using stress–strain measurements, elastic modulus, break elongation, and tensile strength.	Tensile strength tester Atomic force microscopy (AFM)	[72,73,74,76,79]
Degradation kinetics	Understanding culture stability and biophysical properties such as hydrogel elastic modulus are made possible with the degradation kinetics analysis. Changes in mechanics and swelling that may affect cell behaviors such as motility, spreading, and traction force generation is correlated with degradation kinetics. Stability to a certain timescales is useful even for degradable hydrogels mechanical and or enzymatic disruption may require in isolating cells from hydrogels that require kinetic degradation analysis.	Buffer degradation profile, changes in mechanical properties	[72]
Antimicrobial activity	Enables us to understand tissue infections through bacterial surface adhesion and subsequent colonization.	The agar plate method Disc agar diffusion method	[80,81]
Purity	Rather than extracting cells for analysis, some hydrogel studies will require in-situ cell imaging to visualize cells and biomolecules in hydrogels, necessitating knowledge of hydrogel transparency. Neat hydrogel has a high degree of transparency.	UV–Vis Spectroscopy	[72,74]
Porosity	Influence nutrient flux throughout the matrix is studied by the measurement of the diffusion of fluorescently tagged polymers entrapped within the hydrogel. The ability of hydrogel to allow nutrients, oxygen, and metabolic products to diffuse easily into their matrices need to be studied.	SEM, Fluorescence recovery after photo-bleaching (FRAP), DNA electrophoresis	[72,79]
Self-healing activity	Considering the strong penetrability to biological systems, examining the reversible melting process and recrystallization under heating and cooling cycle of hydrogel is essential.	Healing efficiency calculation by tracking optical microscopy.	[82]
Electro stimulated Cell Culture	To examine cell viability through electro stimulating potentials	Fluorescence staining and a MTT assay.	[77]
Animal Experiments	The electro-active hydrogels combined with electrical fields, mimicking the electro-physiological environment in native tissues for proof of concept in skin tissue regeneration.	in vitro biological evaluation	[77]

**Table 3:** Conductivity of human tissue (Siemens per meter (Sm^−1^); copied from Reference [85].

Tissues	Sm^−1^	Tissues	Sm^−1^
Cerebellum	0.10	Pancreas	0.35
C.S.F.	2.00	Prostate	0.40
Cornea	0.40	Small intestine	0.50
Eye humor	1.50	Spleen	0.10
Grey matter	0.10	Stomach	0.50
Hypothalamus	0.08	Stomach contents	0.35
Eye lens	0.25	Tendon	0.30
Pineal body	0.08	Testis	0.35
Pituitary	0.08	Thyroid gland	0.50
Salivary gland	0.35	Trachea	0.35
Thalamus	0.08	Urine	0.70
Tongue	0.30	Blood	0.70
White matter	0.06	Cortical bone	0.02
Adrenals	0.35	Bone marrow	0.06
Bladder	0.20	Cartilage	0.18
Large intestine	0.10	Fat	0.04
Duodenum	0.50	Muscle	0.35
Esophagus	0.50	Nerve (Spinal cord)	0.03
Bile	1.40	Skin	0.10
Gall bladder	0.20	Tooth	0.02
Heart	0.10	Ligament	0.30

**Table 4:** Advantages and disadvantages of different design strategies for preparing conductive hydrogels (copied from Reference [87]).

Design Strategies	Advantages	Disadvantages
In situ polymerization	Barrier-free preparation Uniform polymerization	Potentially cytotoxic unreactive oxidants and monomers Need for chemical synthesis process design
Post-polymerization	Adding conductive materials to synthesized hydrogels Possibility of the conductive coating method	Cytotoxic unreactive oxidants and monomers Additional polymerization step
Composite strategies	Adjustable conductivity No cytotoxic unreactive oxidants or monomers	Non-uniform additive distribution Conductive additive toxicity

## Data Availability

The data used to support the review summary of this paper are included within the article.
